# Simulation of transportation of acute stroke patients in border regions

**DOI:** 10.1038/s41598-024-51959-y

**Published:** 2024-01-19

**Authors:** Peter B. Sporns, Urs Fischer, Mira Katan, Johanna M. Ospel, Alex Brehm, Ioannis Tsogkas, Jessalyn K. Holodinsky, Noreen Kamal, Jens Fiehler, Marios-Nikos Psychogios

**Affiliations:** 1 Department of Neuroradiology, Clinic for Radiology & Nuclear Medicine, University Hospital Basel, 4031 Basel, Switzerland; 2 Department of Diagnostic and Interventional Neuroradiology, University Medical Center Hamburg-Eppendorf, Hamburg, Germany; 3 Department of Neurology, University Hospital of Basel, 4031 Basel, Switzerland; 4 Department of Clinical Neurosciences, Cumming School of Medicine, Hotchkiss Brain Institute, University of Calgary, Calgary, AB Canada; 5 Department of Industrial Engineering, Faculty of Engineering, Dalhousie University, Halifax, NS Canada

**Keywords:** Stroke, Neurology

## Abstract

Determining the optimal transportation for each stroke patient is critically important to achieve the best possible outcomes. In border regions the next comprehensive stroke center may be just across an international border, but bureaucratic and financial hurdles may prevent a simple transfer to the next stroke center. We hypothesized that in regions close to international borders, patients may benefit from an "open border, closed transfer scenario", meaning that patients in whom a large vessel occlusion (LVO) is detected in the primary stroke center will benefit from a transfer to the nearest stroke center offering endovascular thrombectomy—even if this may be across a national border. We used the Swiss-German–French trinational region as an example for a region with several international borders within close proximity to one another, and compared two feasible scenarios; (a) a “closed borders, open transfer” scenario, where the patient is transported to any center in the same country, (b) an “open border, closed transfer” scenario, where patients are always transported to the nearby primary stroke center first and then to the nearest comprehensive stroke center in either the same or a neighboring country and (c) and “open borders, open transfer” scenario. The outcome of interest was the predicted probability of acute ischemic stroke patients to achieve a good outcome using a conditional probability model which predicts the likelihood of excellent outcome (modified Rankin scale score of 0–1 at 90 days post-stroke) for patients with suspected LVO. Results were modeled in a virtual map from which the ideal transport concept emerged. For an exemplary LVO stroke patient in Germany, the probability of a good outcome was higher in an open border, closed transfer scenario than with closed borders, open transfer (33.1 vs. 30.1%). Moreover, time to EVT would decrease from 232 min in the first scenario to 169 min in an open border, closed transfer scenario. The catchment area of the University Hospital Basel was almost double the size in an open border, closed transfer scenario compared to closed borders (1674 km^2^ vs. 2897 km^2^) and would receive transfers from 3 primary stroke centers in other countries (2 in Germany and 1 in France). Stroke patients showed a higher likelihood of good outcome in the “open border” scenarios without transfer restrictions to a specific healthcare system. This probably has implications for stroke treatment in all border regions where EVT eligible stroke patients may benefit from transport to the closest EVT capable center whenever possible, regardless of whether this hospital is located in the same or a neighboring country/jurisdiction.

## Introduction

Determining the optimal transport destination for patients with suspected stroke is of critical importance as the effects of both intravenous thrombolysis (IVT) and endovascular thrombectomy (EVT) diminish over time^1^. Even though optimization of in-hospital workflow has undergone extensive research, prolonged pre-hospital transport time is one of the major factors causing treatment delays and transportation of the right patient, to the right hospital with the right facilities, can still be improved. Previous research on optimal stroke patient transportation has shown that it is context specific and the radius of superiority of the transport strategy changes based on primary and comprehensive stroke centers’ workflow times and proximity of both centers, as well as transport times, and the triaging tool used^2^. It has been demonstrated that initial transport to a primary stroke center (PSC) that is in close proximity to a comprehensive stroke center (CSC) is only beneficial when the PSC is able to achieve a door-to-needle time of equal or less than 30 min^3^ Also, there is a considerable impact of transportation times on the efficiency of each model^4^. All these modelling studies are based on the assumption that there is unrestricted access to both the PSC and the CSC. This, however, is often not true as in regions like Europe where several relatively small countries with different health care systems are close neighbors, the closest CSC may just be across an international border, whereas the next CSC in the same country may be several kilometers away. In general, patients with a suspected large vessel occlusion (LVO) can be initially transferred to the PSC to minimize time to imaging and possible IVT and then get transferred to the nearest CSC only in the same country (closed border scenario) or they get transferred to the closest CSC independent from in which country this CSC is located (open transfer scenario). The open border scenario may decrease the time to EVT and lead to better clinical outcomes compared to the closed border scenario.

Therefore, we hypothesized that in regions close to international borders, patients may benefit from an open border scenario; i.e. clinical outcomes of stroke patients may generally be better if transfer access to the fastest EVT-capable CSC was available. Using the example of the Swiss-German–French tri-border region with our own hospital, the University Hospital Basel as a CSC and the nearby PSCs, we modelled transport times for patients in the nearby Swiss, German and French regions in various transfer scenarios.

## Methods

### Geographic environment

**Figure 1:**
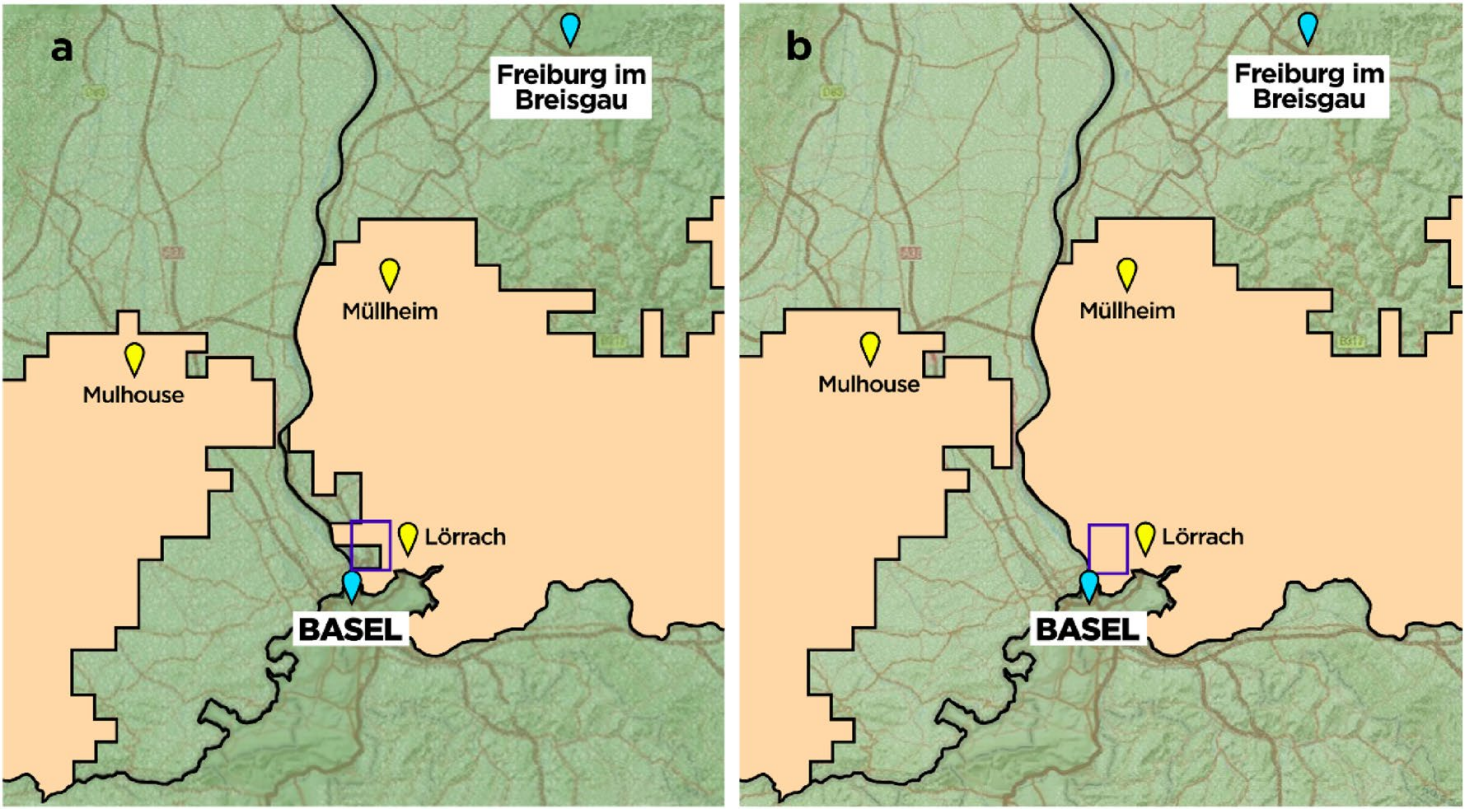
Example of a patient in Germany in the Haltingen/Binzen area (boxed in purple). Closed borders, open transfer ((**a**) left), open borders, closed transfer ((**b**) right). (**a**) A patient in the Haltingen/Binzen area (boxed in purple) is always transported directly to the closest stroke center but only in the same country (“closed border, open transfer”). The closest center to patients in these towns is Lörrach which is a ~ 13 min drive, in a drip and ship transfer scenario patients would be transferred onwards to University Hospital Freiburg for EVT with a travel time of ~ 49 min between centers. The probability p for a good outcome would be 0.3360 for all RACE ≥ 5 patients, 0.3018 for all LVO patients and 0.3484 for all ischemic stroke patients. Estimated onset to thrombolysis in the primary stroke center would be 103 min (60 min onset to ambulance, 13 min travel to PSC, 30 min DTN) and estimated onset to EVT time would be 232 min (above plus 20 min needle to door, 49 min transport, 60 min door to arterial access). All areas which are beige should send RACE ≥ 5 patients to the nearest primary stroke center as outcomes were better if this option was chosen. Green areas in Germany and France near the borders should send RACE ≥ 5 patients directly to the closest comprehensive stroke center (not to PSC first) in this model, mostly due to road conditions such as direct highway access. (**b**) The same patient in the Haltingen/Binzen area is shown in an “open border, closed transfer” scenario. This means that the patient is always admitted to the closest primary stroke center in the same country but can then be transferred to any comprehensive stroke center even if this is in another country. The exemplary patient (boxed in purple) is again transported to Lörrach but according to this model LVO stroke patients would then be transferred onwards to University Hospital Basel for EVT with a travel time of ~ 17 min between centers. The probability p for a good outcome would be 0.3516 for all RACE ≥ 5 patients, 0.3314 for all LVO patients and 0.3726 for all ischemic stroke patients. Estimated onset to thrombolysis in the primary stroke center would again be 103 min (60 min onset to ambulance, 13 min to PSC, 30 min DTN) but estimated onset to EVT time would decrease from 232 min in model A to 169 min in this model. Thus, the same patient would save 63 min until EVT was started. In this second scenario there are still green regions near the border, where patients should be driven directly to a CSC, but not in Switzerland, which seems not reasonable. The reason for this is the hypothesis in this scenario that all German or French patients should first go to a near PSC (see also Fig. 2).

We used the Swiss-German–French trinational region as an example for a region with several international borders within close proximity to one another, in which the difference between open vs. closed borders scenarios can have substantial influence on patient outcome. In reality, there is some degree of international cooperation between the three countries in medical emergencies, meaning that patients can be transported across the border but have to be admitted to a primary stroke center first. This is why we primarily compared two scenarios for this study, namely the “closed borders, open transfer,” scenario (scenario a), which is most common in reality and where the patient is transported to the nearest primary stroke center or comprehensive stroke center but only in the same country. In this scenario a direct transfer to a national CSC or secondary transport to the CSC is also possible (organization methods drip and ship versus mothership). Scenario (b) is an “open borders, closed transfer” scenario, where patients are always transported to the nearby primary stroke center first (has to be in the same country) and then transferred to the nearest comprehensive stroke center in either the same or a neighboring country. We also mapped the not realistic “open borders, open transfer” scenario (scenario c) where a patient with a suspected LVO can travel directly to a CSC across the border, even without stopping at a PSC (also included in Fig. 1a). This scenario is currently not likely to be feasible due to exhausted treatment capacities and financial regulations. We defined a PSC as a hospital that provides IVT 24 h a day, 7 days a week. A CSC is defined as a hospital that provides both IVT and EVT 24 h a day, 7 days a week.

### Predictive model

DESTINE Health is a software which predicts the most optimal first transport destination for patients with suspected stroke due to LVO. The software incorporates data on the decay in probability of excellent outcome over time for both alteplase and EVT treatment, the PPV and false positive diagnostic distribution for LVO screening tools, the drive times from the geographic location to the thrombolysis centres and EVT centres, along with the user entered hospital treatment times. The result is a colour coded map indicating the transport option which yields the highest probability of excellent outcomes^2,5^.

### Modeling parameters

The assumed baseline patient population had a score of ≥ 5 on the Rapid Arterial Occlusion Evaluation (RACE) scale and would thus screen positive for a probable LVO^6^. We assumed that 90% of LVO patients would be treated with EVT and that 80% of patients with LVO or non-LVO occlusions with onset-to-treatment times < 4.5 h would receive IVT.

Based on average times from the German Stroke Registry^7^ and our own published results^8,9^ the following time parameters were used: onset to first medical response time: 30 min (min), ambulance on scene time: 30 min, door to needle time at PSC: 30 min, needle to door time at PSC: 20 min and door to needle time at CSC: 30 min. If a transfer patient arrived at the CSC Basel within 60 min from first imaging at the PSC, it was assumed that the patient did not require reimaging and was transferred directly to the angiosuite, and the door to puncture time would be 30 min. For mothership patients or transfer patients requiring re-imaging door to puncture time was 60 min.

Holding all treatment times constant the delay (or acceleration) in onset to needle time for a closed vs. open-transfer scenario can be calculated as:

$${\Delta }_{onset-needle}=transport\, time\, from\, scene \,to \,PSC-transport \,time\, from\, scene\, to \,CSC$$

Holding all treatment times constant the delay (or acceleration) in onset to groin puncture times for a closed vs. open-transfer scenario can be calculated as:

$${\Delta }_{onset-groin(no \,reimaging)}=\left(transport \,time\, from\, scene\, to \,PSC+needle \,to\, door\, {}_{at}PSC+transport \,time \,between \,PSC\, and \,CSC\right)-(transport \,time\, from\, scene \,to \,CSC+ needle\, to\, door\, {}_{at}CSC$$

$${\Delta }_{onset-groin(reimaging\, required)}=\left(transport\, time \,from\, scene\, to\, PSC+needle\, to\, door{}_{at}PSC+transport\, time\, between\, PSC \,and\, CSC+30 \,minutes\, (reimaging \,time)\right)-(transport \,time\, from \,scene\, to\, CSC+ needle\, to \,door\, {}_{at}CSC$$

### Visualization of the results (transportation maps)

Study results were visualized using DESTINE mapping software (DESTINE Health Inc., Calgary, AB, Canada). To indicate which transport option predicts the best probability of good outcome the maps are color coded: red areas indicate that a “PSC first, then CSC” transport paradigm predicts the highest probability of good outcome, green areas indicate that a “direct to CSC” transport paradigm predicts the best probability of good outcome. All PSCs are depicted as yellow pinpoints and all CSCs are depicted as light blue pinpoints.

## Results

Two scenarios were calculated for two exemplary patients in the three-country border region surrounding the university hospital (University of Basel, Switzerland): the first with “closed borders, open transfer”, the second with “open borders, closed transfer (meaning primary imaging and possible IVT in closest PSC in same country and then transfer to closest CSC in any country)”. Transportation maps were generated for all scenarios (Fig. 1).

Figure 1 shows the example of a patient with a stroke (RACE ≥ 5) in the close-to-border German region Haltingen/Binzen with both models (patient represented by the purple box). A patient with an LVO has a probability of an excellent outcome of 30.1% if (s)he is transported to the closest stroke center but only in the same country (open transfer, closed borders) and 33.1% in a scenario where (s)he is always admitted to the closest PSC in the same country and then transported to the closest CSC capable of performing EVT (open borders, closed transfer). For this exemplary patient, time to thrombolysis would be 103 min for both scenarios but time to EVT would decrease from 232 min with transport to the EVT-capable CSC in Germany to 169 min with transport to EVT-capable center across the border in Switzerland. The beige colored areas in Fig. 1a indicate the regions where a PSC-first transfer should be preferred; in Fig. 1b the beige colored areas indicate better clinical outcomes for the patients with a transfer to a PSC-first and then to the CSC in Basel, as there are no border restrictions in this scenario. A direct to CSC in another country (“open borders, open transfer”) scenario is not depicted in this case, as it would require every possible LVO-suspected patient to be transferred to Switzerland directly, which is not feasible due to capacity reasons.

To model the impact of variation of transfer time in this example, we also calculated outcomes for patients with needle-to-door times of 10 min and 40 min (compared to 20 min in the first example). With a needle-to-door time of 10 min a patient with an LVO would have a probability of an excellent outcome of 30.7% if (s)he is transported to the closest stroke center but only in the same country (open transfer, closed borders) and 33.8% in a scenario where (s)he is always admitted to the closest PSC in the same country and then transported to the closest CSC capable of performing EVT (open borders, closed transfer). With a needle-to-door time of 40 min the probability of an excellent outcome would still be 29.6% and 32.1%, respectively.

The second scenario is the example of a patient with a stroke (RACE ≥ 5) in the close-to-border German region Herten/Rheinfelden. According to this model, a patient with an LVO has a probability of an excellent outcome of 29.9% in an closed borders, open transfer model and 32.8% in an open borders, closed transfer scenario. For this exemplary patient, time to thrombolysis would be 107 min for both scenarios but time to EVT would decrease from 236 min with transport to the EVT-capable CSC within Germany to 173 min with transport to EVT-capable center across the border in Switzerland.

**Figure 2:**
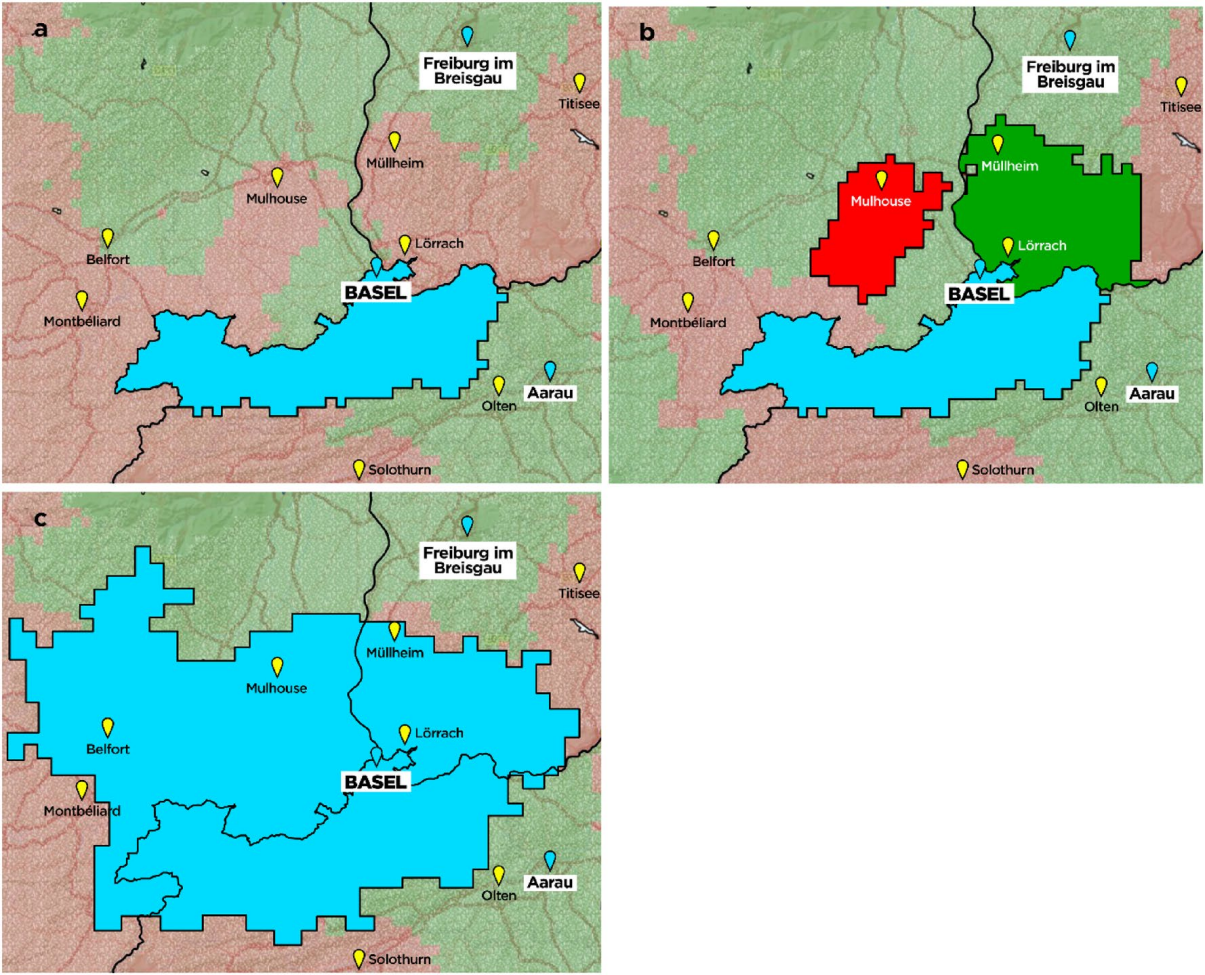
Catchment area of the University Hospital Basel in three different scenarios. (**a**) Catchment area (defined as all patients RACE ≥ 5 in blue) for the University Hospital Basel in an “closed borders, open transfer” scenario, where a stroke patient in Germany, France or Switzerland has to stay in the same country. The size of the mothership catchment is 1674km^2^ and is highlighted in blue. The minimum transport time is 3 min and the maximum is 90 min. Across this entire catchment area, the average probability of good outcomes are for LVO patients 0.3309 and for all ischemic stroke patients: 0.3705. Areas stippled in red will send patients to the nearest primary stroke center (marked in yellow), then to a CSC for thrombectomy and areas stippled in green will send patients directly to the nearest comprehensive stroke center (marked in blue) but both only in the same country. (**b**) Catchment area for University Hospital Basel in an “open borders, closed transfer” scenario. In this model the patient is always admitted to the nearest primary stroke center in the same country and then transferred to the closest comprehensive stroke center in any country. In this model, the CSC Basel would receive transfers from 3 PSCs (2 in Germany, highlighted in green and 1 in France, highlighted in red). In total, the University Hospital Basel would provide EVT services for 2897km^2^ (either by drip and ship or mothership). Across this entire catchment area, the average probability of good outcomes for LVO patients are 0.3300 and 0.3667 for all ischemic stroke patients. Of note, patients in French regions directly neighboring the Swiss border would be transported directly to a comprehensive stroke center in France due to the proximity to a highway. Moreover, regional geographic conditions such as mountains and road conditions in this region do not allow for a more direct way to the PSC so that patients have better outcomes in the model when transferred to French CSC directly. The reason for this is the hypothesis in this scenario that all German or French patients should first go to a near PSC, which is in accordance to the capacity of the CSC in Basel at the moment. Areas stippled in red will send patients to the nearest primary stroke center (marked in yellow) and areas stippled in green will send patients directly to the nearest comprehensive stroke center (marked in blue) but both only in the same country. (**c**) Shows that the catchment area of the CSC Basel (in blue) in an “open borders, open transfer” model would increase to 5693 km^2^. In this model, the patient is always transported either to the closest primary stroke center or directly to the closest comprehensive stroke center without any local or national restrictions. However, this model is not likely to be implemented in the near future due to limited thrombectomy capacities of the University Hospital Basel.

Figure 2 shows the catchment area of the CSC hospital Basel/Switzerland with “closed borders, open transfer” (Fig. 2a) and in an “open borders, closed transfer” (Fig. 2b) scenario. The area in which patients are more likely to have a good outcome when transferred to the CSC increased in an “open border, closed transfer” (1674 km^2^ vs. 2897 km^2^). In an “open border, closed transfer” scenario the CSC Basel/Switzerland would receive transfers from 3 PSCs in other countries (2 in Germany and 1 in France, colors bright red and bright green in 2B). Figure 2c additionally shows that the catchment area of the CSC Basel would increase to 5693 km^2^ in a theoretical “open borders, open transfer” scenario, where patients are always transferred to the nearest PSC or CSC depending on the probability of an LVO and without any national restrictions.

## Discussion

Our results demonstrate that patients in close-to-border regions may have a higher likelihood of a good outcome if stroke treatment is delivered without restrictions to one specific national healthcare system. International co-operations in the tri-national region of Basel are well-established in some healthcare areas, but this is mainly related to chronic conditions and trauma patients. So far, routine transport of stroke patients to nearby hospitals in neighboring countries is not established as standard of care. However, time-critical conditions such as acute ischemic stroke force healthcare policymakers to rethink national restrictions. In regions like Europe, where several relatively small countries are located in close proximity and share multiple borders, this seems even more important than in areas like North America, where countries have only one or few neighboring countries. However, our results are not only applicable to national borders; they can in principal be applied to any geographic area with different healthcare systems. In the United States or in Canada for instance, transport of acute ischemic stroke patients across state borders may sometimes be difficult, and the benefit of “opening the borders” between states for stroke patient transport paradigms may yield similar benefits to those shown in our study.

In the current situation the only feasible option due to healthcare insurance regulations in the different countries and capacity reasons in Basel /Switzerland would be the “closed borders, open transfer scenario”, in which the patient is always transported to the nearest PSC in the same country first and then gets transported to the closest CSC, even if the latter is located across a national border. In contrast, we do not think that an “open borders, open transfer” scenario is likely to be feasible and thus, it was just added for the sake of completeness of this study. Considering the results of the RACECAT randomized clinical trial a longer transportation to the CSC may also be associated with worse outcomes in hemorrhagic strokes partially compensating the positive effect in patients with ischemic stroke due to LVO, a factor that has not been considered in our study^10^.

From a health economics perspective, the shortest transport of patients with proven LVO from a PSC to the CSC may still be cost-effective as it has been shown that any time delay to endovascular stroke thrombectomy reduces quality-adjusted life-years (QALYs) and decreases the economic value of care provided by this intervention^11^: The net monetary benefit per 10 min time to treatment decrease for an average patient in the United States for example is estimated at approximately 10.600 USD^12^. However, cost-effectiveness analyses in such a multi-national context are difficult, since costs for stroke treatment may be different even in neighboring countries such as Switzerland and Germany.

This work has some limitations. First, the results of our model may not be applicable to other regions as there may be an impact of peak traffic periods on calculating driving times in different regions^4^. Second, outcome decay curves used in our model were derived from clinical trials containing highly selected patients from Europe and North America and might not be completely translatable/generalizable to other regions, and to daily clinical practice. Third, the percentage of patients treated with IVT or EVT influences the modeled results and these proportions might be variable in different countries. Fourth, several prehospital stroke severity scales have been developed to identify patients with LVO. In our model, we chose a score of ≥ 5 on the RACE scale for the selection of the patient population. As has been shown before, catchment areas were similar using different screening tools, such as the Los Angeles motor scale or the Cincinnati stroke triage assessment tool; however, the results would be different if a more accurate screening tool for LVO were to become available in the future. Fifth, average time parameters were used from the German Stroke Registry but may be different in other countries and differ for each patient. Finally, we considered neither air transport nor mobile stroke units, nor did we take into account extraordinary traffic congestion, which can be the result of inclement weather, accidents or construction works.

## Conclusion

The results of our study and the generated maps can provide a starting point for the improvement of stroke care in the tri-national region of south-west Germany, north-west Switzerland and south-east France. It becomes evident that the current stroke systems of care do not provide the quickest treatment pathway for stroke patients. Local politicians, insurance companies and treatment providers should discuss and implement more effective pathways in the near future, aimed at improving patient outcomes and not restricted by national borders.

## Data Availability

The data analyzed for the current study were generated with commercially available DESTINE Health software and are available from the corresponding author on reasonable request. No patients were involved in this study. As no patient data were used for this study, institutional review board approval was not sought. The study was conducted in accordance with the Declaration of Helsinki.
